# Supplementary material for: Teenage Blues: Predictors of depression among adolescents in Nigeria
Source: PLoS One. 2024 Apr 17;19(4):e0293995. doi: 10.1371/journal.pone.0293995 (PMC11023510; doi:10.1371/journal.pone.0293995)
Supplement: Attachment — Submitted filename: Reviwer feedback Plosone.docx [file pone.0293995.s002.docx]

Reviewer’s Feedback

Dear Editor,

Thank you for the opportunity to review the manuscript with the title “Teenage Blues: predictors of depression among adolescents in Nigeria”. The manuscript is an interesting read and provides knowledge about the burden depression among adolescent students in Abeokuta, Nigeria. However, there are some drawbacks.

Introduction

Line 96: Full stop is missing at the end of psychosocial functioning.

Methodology

Sampling and sample size: The criteria for selecting the 5 schools out the 39 public schools are not clear, noting that some of these public schools may be relatively different in some socio-demographic/economic characteristics based on where they are sited. Some schools with specific characteristics within this sub population may have been missed by just picking few schools randomly from these numbers. Again, private schools which constitute a significant proportion of this subgroup of adolescents in secondary schools with possibly different characteristics were not included. These factors may significantly affect the generalizability of the results. These I think are major setbacks, though some were mentioned.

Data Analysis: The later version of SPSS, version 23 and above are referred to as IBM SPSS statistics version (.......) as against Statistical Package for Social Sciences (SPSS) for Windows.

Results

Table 3: Correlates of Depressive Disorders

To improve the meaningfulness of the data, those variables that are less than 5 can be merged with closet options, more importantly for: mother's education, domestic violence, family functioning, sex frequency, being bullied. For example, for sexual frequency, most days and Some days can be merged together, FAMILY FUNCTIONING (Highly Dysfunctional and Moderately Dysfunctional Family can be merged together) etc.

Again, the χ2 and df can be removed from text, they do not add any additional information.

Similar adjustment can be made for similar variables in the multivariate regression analyses in Table 4.

Discussion

Line 265: “The reported prevalence in this study is that reported in other studies” This is not clear. Please, recast.
